# Do oral aluminium phosphate binders cause accumulation of aluminium to toxic levels?

**DOI:** 10.1186/1471-2369-12-55

**Published:** 2011-10-12

**Authors:** Ruth Pepper, Neil Campbell, Magdi M Yaqoob, Norman B Roberts, Stanley L-S Fan

**Affiliations:** 1 Department of Renal Medicine and Transplantation, Royal London and St Bartholomew's Hospitals, London, UK; 2 Department of Clinical Biochemistry, Royal Liverpool and Broadgreen University Hospitals, Liverpool, L7 8XP, UK

## Abstract

**Background:**

Aluminium (Al) toxicity was frequent in the 1980s in patients ingesting Al containing phosphate binders (Alucaps) whilst having HD using water potentially contaminated with Al. The aim of this study was to determine the risk of Al toxicity in HD patients receiving Alucaps but never exposed to contaminated dialysate water.

**Methods:**

HD patients only treated with Reverse Osmosis(RO) treated dialysis water with either current or past exposure to Alucaps were given standardised DFO tests. Post-DFO serum Al level > 3.0 μmol/L was defined to indicate toxic loads based on previous bone biopsy studies.

**Results:**

39 patients (34 anuric) were studied. Mean dose of Alucap was 3.5 capsules/d over 23.0 months. Pre-DFO Al levels were > 1.0 μmol/L in only 2 patients and none were > 3.0 μmol/L. No patients had a post DFO Al levels > 3.0 μmol/L. There were no correlations between the serum Al concentrations (pre-, post- or the incremental rise after DFO administration) and the total amount of Al ingested.

No patients had unexplained EPO resistance or biochemical evidence of adynamic bone.

**Conclusions:**

Although this is a small study, oral aluminium exposure was considerable. Yet no patients undergoing HD with RO treated water had evidence of Al toxicity despite doses equivalent to 3.5 capsules of Alucap for 2 years. The relationship between the DFO-Al results and the total amount of Al ingested was weak (R^2 ^= 0.07) and not statistically significant. In an era of financial prudence, and in view of the recognised risk of excess calcium loading in dialysis patients, perhaps we should re-evaluate the risk of using Al-based phosphate binders in HD patients who remain uric.

## Background

The importance of preventing hyperphosphataemia is well established and its management is outlined in the bone and mineral metabolism section of the K-DIGO [1]. The evolution of phosphate binder therapy in patients with chronic renal disease has followed an interesting pattern over the past 35 years. Historically, aluminium salts were used to treat hyperphosphataemia, but safety concerns about accumulation and toxic effects including osteomalacia and encephalopathy meant there was a switch to calcium based binders (carbonate or acetate) [2].

However, the accumulation of aluminium was found in dialysis patients at a time when haemodialysis was conducted against water that might have contained aluminium (concentration depending on the local water) [3,4]. Haemodialysis using Reverse-Osmosis (RO) treated water has been the norm since the late 1990's [5] and it remains to be determined if clinically significant aluminium accumulation occurs when aluminium based phosphate binders are ingested without the confounding factor of aluminium-contaminated dialysis.

All water for haemodialysis at Barts and The London NHS Trust was treated by RO since early 1990's. We contemporaneously recorded the medications of dialysis patients and so are able to identify current patients that were treated with oral aluminium. We quantified aluminium ingested by patients who started HD since Jan 2002 (and therefore never exposed to Al-contaminated dialysis water).

To assess the risk of toxic accumulation of aluminium in these identified patients, we performed a validated low-dose desferrioxamine (DFO) test [6]

## Methods

This study followed guidelines set out by the local Ethics Committee; DFO tests were performed on patients for clinical reasons to determine level of aluminium toxicity in patients with previous exposure.

All patients that started haemodialysis since Jan 2002 and were still on treatment at 1^st ^June 2009 were identified through our electronic patient records. We reviewed and quantified the amounts of aluminium that were prescribed for these patients. The load of aluminium was calculated on the basis that each Alucap™ capsule contained 475 mg of dried aluminium hydroxide (equivalent to 174 mg of aluminium). These patients underwent a low-dose DFO test performed during a 48 hr inter-dialytic period. Briefly, 500 mg of desferrioximine (DFO) was administered intravenously at the end of their dialysis. Pre-DFO and 48 hr post dose (pre-dialysis) aluminium concentrations were measured. Evidence of significant *in vivo *aluminium load was defined if baseline serum aluminium concentration was > 1.0 μmol/L, whilst post DFO levels > 3.0 μmol/L was defined to indicate significant risk of aluminium toxicity. During and after the DFO test, haemodialysis staff were asked to report any adverse events that might be related (with particular emphasis on infections and pyrexia of unknown origins but we did not specifically examine visual acuity).

Formal documentation of residual renal function of haemodialysis patients was not routine practise at our institution. However, for the purpose of this study, we classified patients to anuric if they self reported their RRF as < 200 mls/day.

### Laboratory Methods

Serum Aluminium levels were measured by ICPMS. The instrument used was an XSERIES2 ICPMS (Thermo Fisher, Hemel Hempstead, UK). Ultra pure water [Elga UHQII unit, High Wycombe, UK)] and ICPMS quality reagents were used. The assay standard was multi-element containing 9.27 μmol/L Aluminium and elements Cadmium, Copper, Lead, Manganese, Selenium and Zinc. Control sera was obtained from Bio-Stat Diagnostics, UK. The controls and tests 125 μl (plasma, LiHep) were thoroughly mixed with 5 ml of internal standard/diluent containing 20 ppb Gallium,10 ppb Indium, 10 ppb Rhodium, 5 ppb Scandium in 1% Nitric Acid, 0.2% Propan-2-ol, 0.2% Butan-1-ol and 0.005% Triton X-100. Aluminium mass 27 was monitored with the internal standard ion scandium mass 45. The signal for zero diluent was usually less than 3000 with 90,000 counts for the 9.27 μmol/L standard.

The detection limit for the assay was 0.1 μmol/L and within-batch CV [n = 6] was:- at 0.5 μmol/L 3.7%, 1.1 μmol/L 6.4%, 4.3 μmol/L 5.3%, respectively and the inter-batch CV [n = 13] at 0.4 μmol/L 17%, 1.3 μmol/L 6.0%, 3.9 μmol/L 4.5% and 7.4 μmol/L 4.0%..

### Statistics

This was mostly a descriptive study of the results of DFO tests performed in patients prescribed oral aluminium-based phosphate binders. However, univariate regression analysis was performed to compare the pre-DFO and post-DFO serum aluminium level with the total cumulative prescribed aluminium load. We also compared the increment in serum aluminium level after DFO with the prescribed aluminium load.

## Results

**Figure 1:**
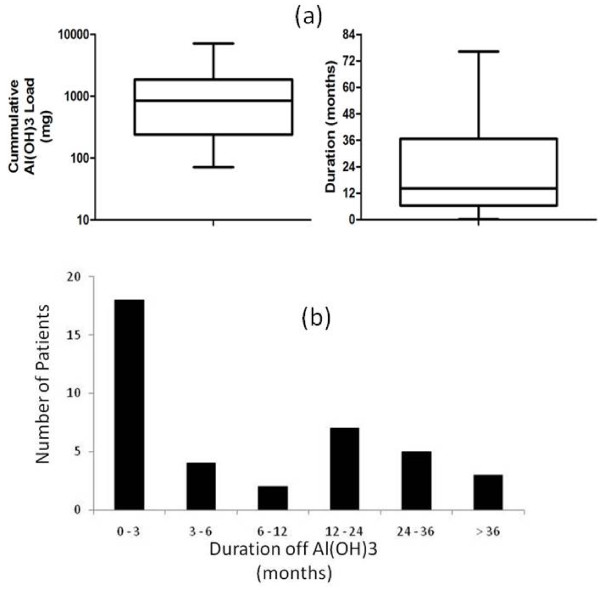
**The amount of aluminium prescribed (load and duration) and the duration off the drug prior to DFO test**.

We identified 39 current haemodialysis patients that fulfilled our criteria of starting HD since Jan 2002 and having prior prescriptions of oral aluminium salts as a phosphate binder. The mean total cumulative dose of aluminium hydroxide was 1.2 kg (range: 71 g - 7.15 kg) whilst the median cumulative dose was 848 g (with 1^st ^and 3^rd ^quartile values of 252 g and 1.85 kg respectively, Figure 1a). The mean (SEM) duration of treatment of the patients was 23.0 months (range 1 - 74 months); with the mean dose of Alucap that was prescribed being 3.5 capsules/day. At the time the DFO test was performed, 13 patients were still taking the drug (mean duration off oral aluminium was 12.9 months) - Figure 1b. There were no reported adverse events related to the DFO tests.

The mean (SEM) age of the patients was 56.5 (2.4) yrs old. None of the patients had been prescribed calcium citrate. Taken as a group, at the time of DFO test, there was little evidence that these patients had hypoparathyroidism; mean (SEM) serum PTH was 48.5 (6.8) pM whilst mean (SEM) Hb concentration was maintained at 9.8 (0.3) g/dl. Thirty-four patients self reported that they had a daily urine output of less than 200 mLs.

**Figure 2:**
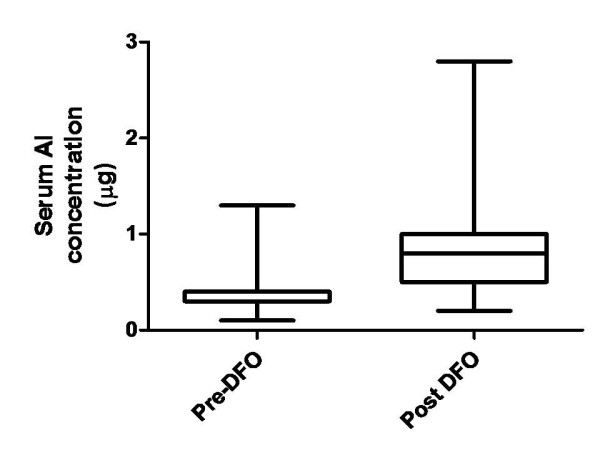
**Serum Aluminium levels of patients exposed to oral Al based phosphate binders pre and post-DFO test**.

Baseline serum Al concentrations were generally low in all patients, Figure 2. No patients had serum Al levels above 3.0 μmol/L and only 2 patients had concentrations above 1.0. These 2 patients had cumulative doses of 1.36 kg and 1.85 kg and both were anuric.

The mean (SEM) baseline Al concentration was 0.39 (0.04) μmol/L. Only 1 patient who had sickle cell disease had a concomitant serum ferritin concentration > 1000 μg/l. This patient had a baseline serum Al concentration of 0.1 μmol/L and no clinical evidence of Al Bone Disease (PTH was 31.8 pmol/L and Alk Phos was 5.8 times the upper limit of normality).

The post DFO serum Al concentration increased from a mean (SEM) of 0.39 (0.04) to 0.85 (0.08)μmol/L as would be expected (p < 0.00001), Figure 2. Nevertheless, none reached the threshold of > 3.0 μmol/L although 12/43 patients had a level > 1.0 μmol/L. Neither of the 2 patients that had evidence of significant baseline Al concentrations (baseline Al level > 1.0 μmol/L) had greater than 3-fold increments in Al level after DFO (incremental rises were from baselines of 1.3 and 1.2 μmol/L to 2.4 and 2.8 μmol/L respectively).

**Figure 3:**
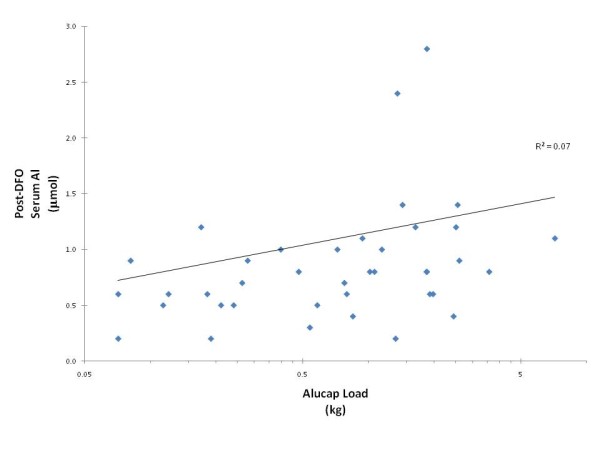
**Correlation between the total cumulative Al (in kg) ingested by patients and their 48 hr post DFO serum Al concentration**.

When we compared the post DFO serum Al concentrations with the cumulative Al load that was prescribed to the patient, we found there was no statistically significant correlation (p = 0.10, Figure 3). Any relationship that did exist was weak (R^2 ^= 0.07).

## Discussion

It has now been almost 2 decades since changes in clinical practises have led to aluminium phosphate binders being replaced by other binders and reverse osmosis (RO) water being used for the preparation of dialysate fluid. High serum aluminium levels are now rare in the dialysis patients [7].

However, aluminium-based binders are highly effective at binding phosphate and causes relatively mild gastro-intestinal discomfort. Fears of re-introducing aluminium toxicity remains despite the fact that many believe aluminium-contaminated dialysate water were a greater contributor to the historic problem than oral phosphate binder usage; in an epidemiological survey of dialysis units, concentration of aluminium in dialysate water correlated with the incidence of both aluminium bone disease and encephalopathy [8].

We have attempted to gain insight into the whether oral intake of aluminium hydroxide will cause significant toxicity by studying patients that were prescribed significant amounts as part of their clinical care.

All baseline serum Al concentrations of patients tested showed levels that were below the threshold that would indicate *potentially *toxic Aluminium levels (> 3.0 μmol/L). However, we know that defining a baseline serum aluminium concentration threshold to diagnose toxic aluminium accumulation is difficult [9] not least because aluminium can be sequestered in bone. Thus, in order to determine total body Al load, we decided to perform DFO tests on all at-risk patients. To maximise sensitivity, we used a post-DFO Al threshold of > 3.0 μmol/L to denote patients at risk of "high total body accumulation". There are different DFO protocols in the literature with doses ranging from 5 to 80 mg/kg [10]. To maximise sensitivity, we wanted to give a larger DFO dose than proposed by D'Haese et al [11] (5 mg/kg), but felt the risk of high dose DFO (30-80 mg/kg) to be unwarranted in a screening program. We therefore decided to use 500 mg- a dose validated against bone biopsies [6] to show a false positive rate of 11% and a false negative rate of 0% (i.e. a negative DFO test had a 100% predictive value). It is particularly reassuring that all patients in our cohort had a negative DFO test.

Although the number of patients studied is small, it is interesting that there were no statistically significant correlations between the serum aluminium concentrations (pre-, post- or the incremental rise after DFO administration) and the total amount of aluminium ingested (although this may because of our small study size). However, even if a statistically significant correlation were to be found in a very large study, we still argue that it would not be clinically significant as our R^2 ^value was only 0.07. We suggest that the aluminium load coming from phosphate binders is likely to be dwarfed by other sources such as drinking water and foods.

We identified 7 patients (18%) that had EPO resistance as defined by by Greenwood et al; Hb < 10.0 g/dl and EPO dose > 23,000 IU/wk [12]. This was higher than the 5% reported in the DOPPS survey that included US and European patients, but, clear alternative reasons could be found in 2 (sickle cell disease and highly inflamed at time of blood sampling) whilst tunnelled dialysis catheters were present in 4 other patients. Moreover, there are many confounders (our patients were likely to be prescribed aluminium because of poor phosphate control and thus EPO resistance may be secondary to hyperparathyroidism rather than Aluminium toxicity).

We interpret our results to suggest that the use of aluminium-based phosphate binders in the era of RO-treated dialysis water appears to be safe. However, our study has many limitations that need to be acknowledged. Many patients were prescribed oral aluminium over a long period of time and we did not collect contemporaneous data about their residual renal function. We determined if patients were anuric at the time of DFO testing, but this would not have identified patients that recently became anuric (but who were uric for the majority of their treatment period). Secondly, interpretations of our results are hampered by the lack of a "control" group. These would have been matched HD patients who had never been prescribed aluminium-based phosphate binders. Thirdly, not all patients were still taking oral aluminium at the time of their DFO test. However, the aluminium has a very long biological half-life and DFO can mobilise the bone stores. Another limitation of our study is the small size and the absence of bone biopsy data. Finally, this was a study of patients that survived after being prescribed aluminium. It is possible that we could not find toxic levels because of selection bias (patients with aluminium toxicity would have died). However, we think this is unlikely; there were no reported cases of suspected aluminium toxicity in our unit. Moreover, even if selection bias existed, we should still have found a significant correlation between aluminium load and DFO results.

In an era of financial prudence, Mudge et al have re-evaluated the risk of using aluminium based phosphate binders in haemodialysis patients who remain uric [13]. We found DFO testing was simple to conduct on a haemodialysis unit and well-tolerated by the patients. The sensitivity and specificity of DFO test are superior to random serum aluminium concentrations for the diagnosis of aluminium bone disease and as an indicator of total body load. We did not perform specific ophthalmologic tests but we were confident that there were no increased incidences of infections or haemodynamic instability related to DFO administration. We believe that DFO testing should be used more frequently if the use of aluminium hydroxide is increased.

## Conclusions

Although this is a small study, oral aluminium exposure was considerable. Yet no patients undergoing HD with RO treated water had evidence of Al toxicity despite doses equivalent to 3.5 capsules of Alucap for 2 years. The relationship between the DFO-Al results and the total amount of Al ingested was weak (R^2 ^= 0.07) and not statistically significant. In an era of financial prudence, and in view of the recognised risk of excess calcium loading in dialysis patients, perhaps we should re-evaluate the risk of using Al-based phosphate binders in HD patients who remain uric.

## Competing interests

The Renal Unit at Barts and The London NHS Trust have received research grants from Genzyme Corporation. SLF has received lecture fees and travel bursaries to attend conferences from Genzyme Corporation.

## Authors' contributions

RP & NC were the researchers who collected the data and performed the DFO tests. MMY contributed to the design of the study and helped with the manuscript. NBR contributed to the design of the study and performed the aluminium analyses. SLF designed and supervised the study.

All authors read and approved the manuscript.

## Pre-publication history

The pre-publication history for this paper can be accessed here:

http://www.biomedcentral.com/1471-2369/12/55/prepub
